# A New Solid-State Proton Conductor: The Salt Hydrate Based on Imidazolium and 12-Tungstophosphate

**DOI:** 10.1021/jacs.1c06656

**Published:** 2021-08-18

**Authors:** Anna Martinelli, José M. Otero-Mato, Mounesha N. Garaga, Khalid Elamin, Seikh Mohammad Habibur Rahman, Josef W. Zwanziger, Ulrike Werner-Zwanziger, Luis M. Varela

**Affiliations:** † Department of Chemistry and Chemical Engineering, Chalmers University of Technology, SE-41296 Gothenburg, Sweden; ‡ Grupo de Nanomateriais, Fotónica e Materia Branda, Departamento de Física de Partículas, Universidade de Santiago de Compostela, Campus Vida s/n, E-15782 Santiago de Compostela, Spain; § Department of Chemistry, Dalhousie University, Halifax, Nova Scotia B3H 4R2, Canada

## Abstract

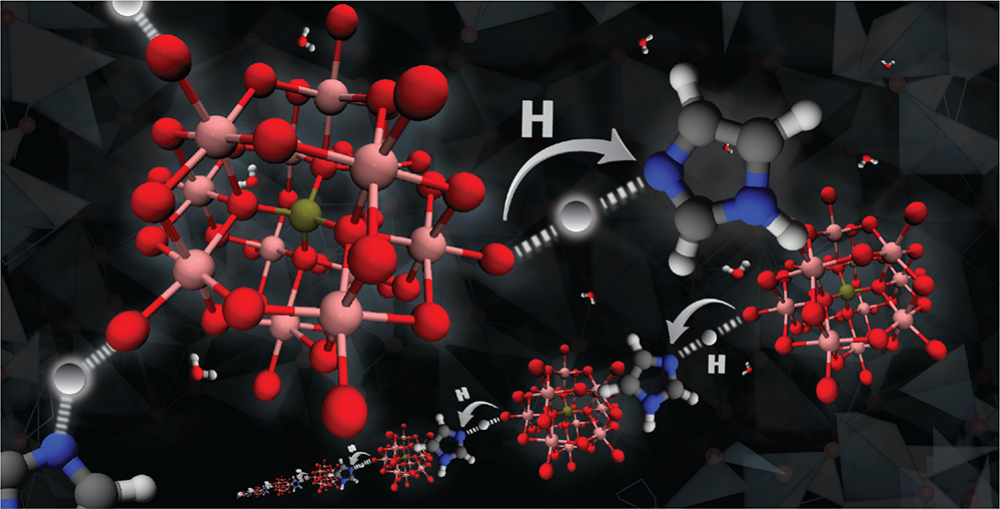

We report the structure and charge transport properties of a novel solid-state proton conductor obtained by acid–base chemistry via proton transfer from 12-tungstophosphoric acid to imidazole. The resulting material (henceforth named Imid_3_WP) is a solid salt hydrate that, at room temperature, includes four water molecules per structural unit. To our knowledge, this is the first attempt to tune the properties of a heteropolyacid-based solid-state proton conductor by means of a mixture of water and imidazole, interpolating between water-based and ionic liquid-based proton conductors of high thermal and electrochemical stability. The proton conductivity of Imid_3_WP·4H_2_O measured at truly anhydrous conditions reads 0.8 × 10^–6^ S cm^–1^ at 322 K, which is higher than the conductivity reported for any other related salt hydrate, despite the lower hydration. In the pseudoanhydrous state, that is, for Imid_3_WP·2H_2_O, the proton conductivity is still remarkable and, judging from the low activation energy (*E*_a_ = 0.26 eV), attributed to structural diffusion of protons. From complementary X-ray diffraction data, vibrational spectroscopy, and solid-state NMR experiments, the local structure of this salt hydrate was resolved, with imidazolium cations preferably orienting flat on the surface of the tungstophosphate anions, thus achieving a densely packed solid material, and water molecules of hydration that establish extremely strong hydrogen bonds. Computational results confirm these structural details and also evidence that the path of lowest energy for the proton transfer involves primarily imidazole and water molecules, while the proximate Keggin anion contributes with reducing the energy barrier for this particular pathway.

## 1 Introduction

**Figure 1:**
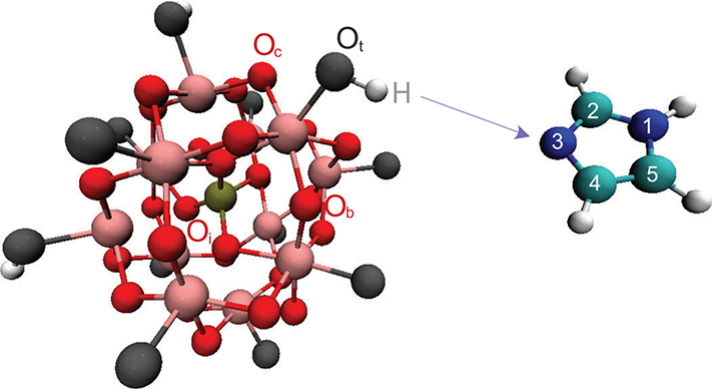
Atomic structure and atom labeling of (left) the Keggin unit of the 12-tungstophosphoric acid H_3_WPA and (right) imidazole in its neutral form. In the Keggin structure, a distinction is made between internal (O_i_), terminal (O_t_), bridging (O_b_) and corner shared (O_c_) oxygen atoms. On imidazole, the hydrogen atoms are at positions N^1^, C^2^, C^4^, and C^5^, while N^3^ is the proton acceptor site.

Achieving high proton conductivities in the solid state is one of the biggest challenges in materials science.^1,2^ In this context, heteropolyacids have attracted considerable attention because of their unique structure and the record proton conductivity among solids, that is, up to 0.18 S cm^–1^ at room temperature for the 12-tungstophosphoric acid hydrate.^3^ Since then a number of solid electrolytes based on heteropolyacids have been reported, which have good potential for use as solid catalysts and in different electrochemical devices including the intermediate temperature H_2_/O_2_ fuel cell. The strongest among solid acids is 12-tungstophosphoric acid, which adopts the so-called Keggin structure with one central P atom surrounded by four O atoms, that are connected to 12 corner shared WO_3_ units, resulting in the molecular formula H_3_[P(W_3_O_10_)4] · *n*H_2_O (which we here abbreviate as H_3_WP), see also Figure 1. The precise location of the acidic protons (H^+^) on the surface of the Keggin anion in the anhydrous state has been a subject of debate, although it seems that terminal (O_t_) and bridging (O_b_) oxygens are the preferred sites.^4^ By contrast, in the hydrated state, protons are located in the water phase as hydronium (H_3_O^+^) or Zundel-like (H_5_O_2_^+^) ions.^5,6^

One drawback of heteropolyacids is the dramatic dependence of proton conductivity on the hydration level, which in turn is a function of temperature and relative humidity. As an example, proton conductivity decays of four or five decades have been regularly reported when the number of coordinating water molecules decreases from 21 to 6.^5^ To circumvent these limitations, novel structures based on the (partial) substitution of the acidic protons with metal cations,^7−10^ or on the incorporation of the heteropolyacid in mesoporous membranes,^11^ have been investigated as alternative, more stable solid-state proton conductors. Moreover, the degree of hydration in these solid acids influences the mechanism of proton transport, which varies from mainly vehicular for a high number of water molecules to Grotthuss for the pseudoanhydrous or totally anhydrous state.^12^ Whether the former or the latter mechanism dominates can be determined from the activation energy of charge transport, *E*_a_, which is of the order of a few 0.1 eV in the case of Grotthuss-like proton transfer through extended hydrogen bonds. In this regard, density functional theoretical (DFT) calculations have revealed that for the case of intramolecular proton transfer events (i.e., those occurring over the surface of one Keggin unit, and not between two different Keggin units), the presence of only one molecule of water bridging over two adjacent oxygen atoms reduces *E*_a_ by 1 order of magnitude, from ∼1.1 to ∼0.11 eV.^12^ In addition, the uptake of even a tiny amount of water, strongly associated with the heteropolyacid clusters, can greatly enhance the transport of protons.^13^ However, this enhancement occurs at the cost of a considerable reduction of thermal and electrochemical stability, which can be solved resorting to protic ionic liquids that are also capable of sustaining high degrees of proton conduction. However, to the best of our knowledge, tuning the conductivity and stability of heteropolyacid-based solid proton conductors with an ion-rich aqueous phase—in a way analogous to that of water-in-salt mixtures^14^—has not been previously attempted.

In this paper, we fill this void by reporting a new proton conducting material obtained via the acid–base chemistry by proton transfer from the 12-tungstophosphoric acid to the base imidazole, the latter being the precursor of a canonical family of well-known ionic liquids. The material that we report is a salt consisting in Keggin anions (WP^3–^) and imidazolium cations (Imid^+^); it has never been reported before and is a material that bridges the gap between proton conducting solid acids and ion conducting salt hydrates. Related materials to this new salt are proton conducting salts formed by proton transfer from a carboxylic acid to imidazole^15^ and those obtained by substituting the acidic protons in 12-tungstophosphoric acid hydrate with metal cations.^7,8,10^ Compared to previously proposed, related materials, the Imid_3_WP hydrate shows superior proton conducting properties despite the lower degree of hydration as well as an improved thermochemical stability. This behavior is rationalized by the crystalline structure adopted, the nature of hydrogen bonds established within the ionic network, and the rotational dynamics of the charge carrying molecules. Both computational and experimental methods are employed to fully characterize this new salt hydrate.

## 2 Experimental Section

### Materials

The 12-tungstophosphoric acid (chemical formula: H_3_PW_12_O_40_; reagent grade; molecular weight: 2880.05 g/mol in the anhydrous state; abbreviated as H_3_WPA in this paper) was purchased from Sigma-Aldrich and was used as received. H_3_WPA is always received in the hydrated form and typically contains 6 water molecules per Keggin unit. Imidazole (chemical formula: C_3_H_4_N_2_; purity above 99%; molecular weight: 68.08 g/mol) was purchased from Sigma-Aldrich and was used as received. Imidazole is in the crystalline form at room temperature but dissolves easily in water. The new salt (abbreviated in this paper as Imid_3_WP) was prepared by mixing 3 mol of imidazole with 1 mol of H_3_WPA (i.e., in a molar ratio that matches the number of acidic protons per unit formula) to maintain charge balance. In practice, 0.5 g of imidazole was added to an aqueous solution of 12-tungstophosphoric acid (7 g in 4 mL of distilled water) at 50 °C and under magnetic stirring. After 30 min of stirring, the milky solution was put aside and stored at ambient conditions (i.e., 24 °C and ambient pressure) to let the excess water evaporate very slowly. After 2 weeks, white colored and mildly translucent crystals had formed with a minimal amount of water left. Thermogravimetric analyses (TGA) revealed that the amount of structural water corresponded to ∼4 water molecules per structural unit, indicating that this salt accommodates less water than similar salts previously investigated. The molecular structure and the atom labeling of the reagents, that is, the base imidazole and the 12-tungstophosphoric acid, are reproduced in Figure 1.

### Thermal Analysis

Differential scanning calorimetry (DSC) measurements were performed using a TA Instruments Q1000 DSC. A sample with a mass between 5 and 9 mg was encapsulated inside a hermetic aluminum pan. The sample was first cooled from 40 °C to −180 °C at a cooling rate of 30 °C/min and thereafter heated to 300 °C with a heating rate of 10 °C/min, under the flow of nitrogen. This procedure was repeated twice.

### Vibrational Spectroscopy

Infrared spectra were collected with a PerkinElmer spectrometer using the attenuated total reflection mode and placing the powder over a single reflection diamond crystal. For each sample, 32 scans were averaged, thus achieving a nominal spectral resolution of 2 cm^–1^. The full spectral range 400–4000 cm^–1^ was investigated. Raman spectra were recorded with an InVia Reflex spectrometer purchased from Renishaw, using the 532 nm line of an Ar^+^-ion laser as the excitation source, which together with a 2400 grooves/mm grating gives a spectral resolution better than 1 cm^–1^. The laser power was set to 20 mW at the source, and spectra were the result of 2 accumulations with a duration of 10 s each. Temperature-dependent Raman spectra were recorded from 25 to 140 °C, using a Linkam Cell (model THMS600) and letting the sample equilibrate at each temperature for 10 min before collecting the Raman spectrum. When a peak fit procedure was employed (e.g., in the region of the O–H bending modes of water or the W=O stretching modes of the Keggin ion), a combination of Lorentzian functions along with a linear background was used.

### Solid-State NMR

One-dimensional (1D) ^1^H and two-dimensional (2D) ^31^P{^1^H} HETero nuclear CORrelation (HETCOR) NMR spectra were recorded, and ^1^H *T*_1_ and *T*_2_ relaxation times were measured on a 400 MHz Bruker Avance DSX NMR spectrometer operating at a magnetic field of 9.4 T (^1^H and ^31^P Larmor frequencies of 400.23 and 162.01 MHz, respectively) using a 2.5 mm double resonance probehead. The ^1^H 90° pulse was set to 4.2 μs. The ^1^H magic angle spinning (MAS) spectrum was collected over 64 transients with 10 s recycling delay by spinning the sample at 25 kHz. The ^31^P{^1^H} cross-polarization (CP) was achieved by crossing through the Hartmann–Hahn condition via RAMP CP with the contact time of 800 μs. A radio frequency (RF) field strength of 50 kHz (ramping down) and 33 kHz was used for ^1^H and ^31^P, respectively, to get a good CP condition. A ^1^H RF field strength of 75 kHz was used for the SPINAL decoupling during acquisition. The ^31^P{^1^H} HETCOR NMR experiment was performed at 15 kHz MAS rate. The spectrum was acquired over 160 scans for each of 80 slices in F1 dimension with 3 s recycling delay. ^1^H *T*_1_ and *T*_2_ relaxation times were measured by using saturation recovery and Carr–Purcell–Meiboom–Gill pulse sequence, respectively, in the temperature range between 295 and 330 K, at 20 kHz MAS speed. The ^1^H chemical shifts were externally referenced to that of adamantane. The ^31^P chemical shifts were calibrated by using the ^1^H chemical shift reference.

### Impedance Spectroscopy

The temperature dependence of the ionic conductivity was measured by dielectric spectroscopy using a Novocontrol broadband dielectric spectrometer in the frequency range 10^–1^–10^7^ Hz. The sample was placed between two gold-plated electrodes using a Teflon spacer (diameter: 7 mm; thickness: 0.25 mm) and loaded into a Quatro Cryosystem temperature control unit. Data were collected in steps of 10 °C, thermally equilibrated for 20 min at each temperature, in three ramp intervals: from −50 to 200 °C, from 190 °C to −50 °C, and from −40 to 200 °C, respectively. A steady flow of nitrogen gas was supplied to the sample holder in order to maintain a dry atmosphere. The dc conductivity was extracted from the low-frequency plateau in the frequency-dependent plot.

### X-ray Diffraction

All solid samples (in powder form) were analyzed with the X-ray powder diffraction (XRPD) method. XRPD measurements were carried out at ambient temperature using a Bruker AXS D8 ADVANCE VARIO powder diffractometer equipped with a curved germanium primary monochromator (CuK_α1_ = 1.5406 Å) and a solid-state LynxEye detector. For all samples, scans were preformed covering the 2θ range 2.25°–75°/90°, with 0.034 step size, 1–4 s per step and a total scan time of 1–2 h. Approximately 200 mg of sample was ground for about 5 min in an agate mortar and pestle to achieve an homogeneous powder. The measurements were made in reflection mode (Bragg–Brentano geometry) on powders kept in PMMA sample holder rings with an internal diameter of 20 mm. The collected patterns were imported in the evaluation software package DIFFRACplusEVA with SEARCH function, where phases can be identified using the PDF (ICDD, 2015) database and The Cambridge Structural Database. Within these databases we could not find any structural match.

### Scanning Electron Microscopy

Scanning electron microscopy (SEM) images were collected using a LEO Ultra 55, operating at an accelerating voltage of 5 kV. All images were acquired in the secondary electron imaging mode to reveal surface structures.

### Surface Analysis

Nitrogen sorption analyses were performed using a TriStar 3000 instrument from Micromeritics Instrument Corporation. The pore size distribution was calculated according to the Barrett–Joyner–Halenda method,^16^ whereas the surface area was obtained using the Brunhauer–Emmett–Teller method.^17^ The pore volume was calculated using a single point adsorption value at the relative pressure (*p*/*p*_0_) of 0.990. These analyses reveal a surface area of 1.96 m^2^/g and a pore volume of 0.009 cm^3^/g, typical of a very compact nonporous material.

### Computational Methods

For a better characterization of the molecular structure of these systems, atomistic molecular dynamics (MD) simulations of Imid_3_WP·*n*H_2_O were performed using Gromacs 2019^18,19^ and the OPLS-AA force-field.^20^ We simulated different hydration states setting *n* to values from 2 to 20, with increments of 2. The SPC model was used to simulate water molecules, that is, a three-site rigid model whose topology is included in Gromacs. The topology parameters for the imidazolium cations were adapted from the ones of 1-ethyl-3-methylimidazolium (EMIM)^21^ which were tested and used in previous studies.^22^ The Lennard-Jones (LJ) parameters were taken from the OPLS-AA force-field, and the partial charges were calculated with the CHELPG scheme^23^ in a DFT calculation using Gaussian 16 software.^24^ We used the basis set 6-311G(d,p),^25,26^ and the exchange–correlation functional was the hybrid functional Becke, three-parameter, Lee–Yang–Parr.^27,28^ Keggin anions were parametrized as rigid molecules using the *virtual*_*sites* option in Gromacs that allows to define the position of the WP atoms referred to a rigid tetrahedron formed by four virtual atoms with the correct mass to reproduce the anion’s mass and moment of inertia. These virtual atoms do not interact with the rest of the particles in the system. Then, the LJ parameters for the real atoms were extracted from ref (29), and the partial charges were calculated in the same way as for the imidazolium cations. The initial configurations for the simulations were obtained by packing randomly 100 Keggin anions, 300 imidazolium cations, and the corresponding amount of water molecules (from 200 to 2000, with increments of 200) to match the hydration state (reported as *n*), using the software Packmol.^30^ The corresponding amount of water is introduced in this box with the *solvate* command in Gromacs. Once the initial configuration was obtained, a short stabilization run of 200 ps with a time step of 0.2 fs in the NVE ensemble, followed by another identical stabilization but in the NVT ensemble using the V-rescale thermostat at room temperature, were performed. Then, a new stabilization run in the NpT ensemble using the Parrinello–Rahman barostat at 1 atm of pressure of 1 ns long but now with 1 fs of time step (which is also used in the following steps) was performed. The resulting stable configuration is used as input for an annealing run, where the system is heated up from room temperature to 500 K during 500 ps. Then, it is simulated at this temperature during 4 ns and finally cooled down to room temperature during 500 ps. This process allows the system to explore a wider area of its energy landscape and find configurations that minimize the energy. Finally, a stabilization run of 10 ns at room temperature and 1 atm precedes a production run 10 ns long, recording atomic positions and velocities every 0.5 ps. The data from this production run were used for the structural analysis. Proton transfer is also studied by means of DFT simulations, calculating the energy barrier that a proton must overcome to move from the donor to the acceptor species. The barrier height is calculated as the difference between the energy of the optimized atomic configuration with the proton bonded to the donor species, which is the imidazolium cation in all the studied situations, and the configuration with the excess proton forming a hydronium cation placed in between the donor and the acceptor. The initial spatial atomic configuration of the pair used for this calculation was extracted from the MD simulations. Then, a geometry optimization of these configurations was performed before the energy calculation, using the same parameters as for the calculation of the partial charges.

## 3 Results and Discussion

### Acid–Base Chemistry

**Figure 2:**
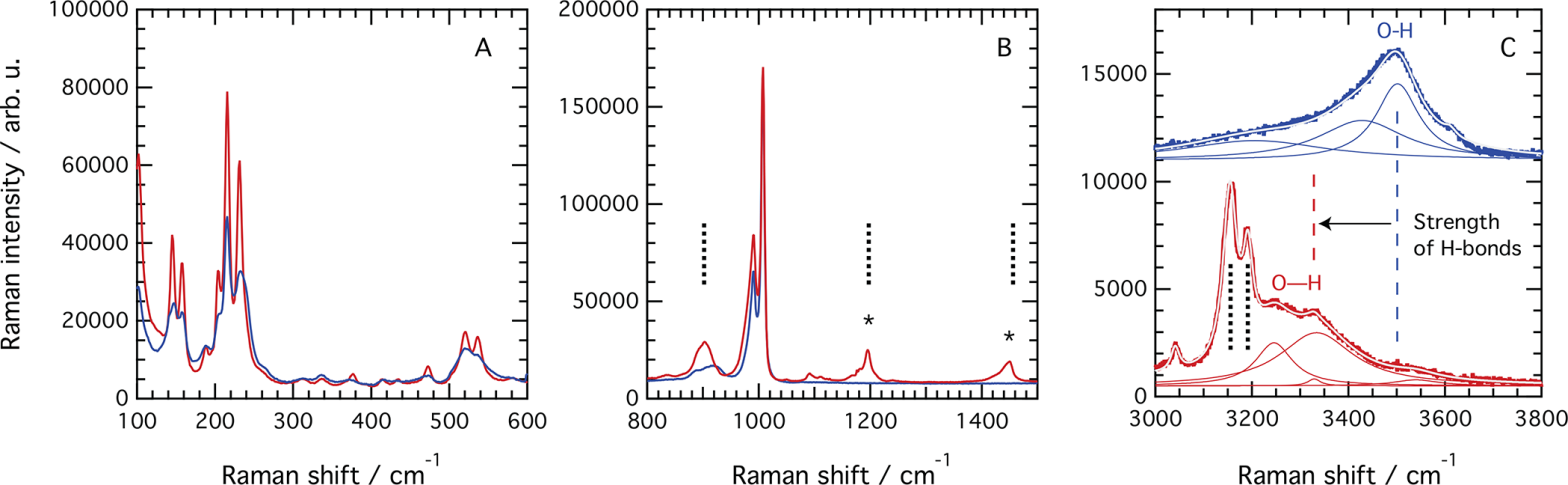
Raman spectra recorded at room temperature for H_3_WPA (blue) and Imid_3_WP (red) in the low-frequency region (A), in the midfrequency region (B), and in the high-frequency region (C). In (B and C), vertical black dashed lines indicate the position of some vibrational modes calculated (after scaling) for protonated imidazole (i.e., for the imidazolium cation) as presented in ref (33). In (B), asterisks indicate vibrational modes arising from the imidazolium cation. In (C), the Raman spectrum of H_3_WPA has been vertically offset for clarity, together with the respective fitting components and the fit result. The fit results for both H_3_WPA and Imid_3_WP are shown as a white curve superimposed to the experimental data.

The salt obtained by reacting 3 mol of imidazole with 1 mol of 12-tungstophosphoric acid, according to the chemical reaction 1H_3_WPA + 3Imid →1WP^3–^ + 3HImid^+^, consists of imidazolium cations and 12-tungstophosphate anions, plus some water of hydration as will be discussed below. This is clear from the Raman spectrum recorded at room temperature for the product material (Imid_3_WP, or Imidazolium tungstophosphate) shown in Figure 2, which reveals the framework and W–O–W bending modes (Figure 2A), the W=O and P–O stretching modes (Figure 2B), and the C–H and O–H stretching modes (Figure 2C), respectively. A complete assignment of the observed vibrational modes can be found in Table 1. In the lower frequency range of the Raman spectrum, that is, below 400 cm^–1^, the vibrational modes attributed to W=O_t_ deformation and to vibrations of the entire framework^32^ appear sharper and more intense in Imid_3_WP than in the reference material H_3_WPA (Figure 2A). This indicates a higher degree of crystallinity and local order and has previously also been observed for salts obtained by substitution of the three protons in H_3_WPA by larger potassium cations.^7^ In Figure 2B, two distinct features are observed at 1194 and 1448 cm^–1^, which are present only in the protonated form of imidazole.^33^ By comparison, unprotonated imidazole shows a richer spectrum in the region 1100–1500 cm^–1^ due to a lower molecular symmetry and thus a higher number of vibrational modes (Figure S1 in the SI file). The theoretically calculated spectrum of imidazolium also predicts a strong vibrational mode at 904 cm^–1^ (which has been observed in infrared but not in Raman spectra) and another polarized mode at 920 cm^–1^. Both are assigned to ring deformation modes.^33^ The intensity of these modes is expected to be approximately 10 times lower than that of the modes observed at 1448 and 1194 cm^–1^, which is not the case for the Raman spectrum of Imid_3_WP recorded in this work. This indicates that the narrower and more intense spectral feature at ∼900 cm^–1^ (red trace in Figure 2B) is due to an intrinsically different local structure of the Keggin ion as compared to the case of H_3_WPA (blue trace in Figure 2B), rather than to interferences from vibrational modes related to imidazolium.

**Table 1:** Observed Raman Active Vibrational Modes and Proposed Assignment

frequency (cm^–1^)	assignment	ref
Keggin Anion		
215	W=O deformation, framework vibr.	(32)
230	W=O deformation, framework vibr.	(32)
520	O_i_–P–O_i_ asym. deformation	(7)
536	W–O_c_–W sym. stretch	(7, 32, 62)
903	W–O–W asym. stretch	(62)
920	W–O_b_–W sym. stretch	(62)
980	P–O_i_ sym. stretch	(40)
990	W=O_t_ asym. stretch	(40, 62)
1007	W=O_t_ sym. stretch	(40, 62)
Imidazolium Cation		
3250	N–H stretch	(34)
3154	C^2^–H stretch	(33)
3191	C^4,5^–H stretch	(33)
1450	ring 3 deformation	(33, 34)
1216	ring 5 deformation	(33, 34)
922	ring 6 deformation	(33, 34)
Water		
3100–3700	O–H stretch	
1550–1750	O–H bending	

The fact that the Imid_3_WP compound includes imidazolium cations is also confirmed in the high-frequency region of the Raman spectrum, which reveals a perfect match between the frequencies of the C^2^–H and C^4,5^–H stretching modes calculated for imidazolium^33^ and those experimentally measured in this work (Figure 2C). Hence, while in the 12-tungstophosphoric acid hydrate protons are in the water phase forming hydronium (H_3_O^+^) or Zundel-type cations (H_5_O_2_^+^),^7,8,12,35−37^ in the ionic compound investigated here, the protons are completely transferred to imidazole. (The fact that water of hydration in Imid_3_WP does not contain hydronium or Zundel-type cations is also shown by the infrared spectrum in the frequency range of H–O–H bending modes, where a single bending mode at 1637 cm^–1^ is observed that is attributed to neutral molecular water. The presence of H_3_O^+^ or H_5_O_2_^+^ species in the H_3_WPA hydrate, on the other hand, is reflected by an additional mode at 1720 cm^–1^, see Figure S2 in the SI file.) This is in full accordance with the acid–base chemistry involved, since the Δp*K*_a_ of the reaction, estimated as p*K*_a_^imidazolium^ – p*K*_a_^H3WPA^, is high enough (i.e., larger than 6.95 – (−13) = 19.95)^38^ to predict a complete proton transfer from the acid to the base.^39^ Very importantly, the Raman spectrum recorded at room temperature for Imid_3_WP also reveals the chemical integrity of the Keggin anion, since the strongest and characteristic vibrations typically found at ∼1007, ∼990, ∼980, and ∼900 are all present with their expected relative intensities (Figure 2B). By contrast, the Keggin anion is unstable at nonstoichiometric ratios of imidazole (as in, e.g., the Imid_4.5_WP salt shown in Figure S3 of the SI file), which is congruent with previous findings that the Keggin structure is stable only at very acidic conditions, typically at pH values lower than 2.^40^

### Strongly Hydrogen-Bonded Water of Hydration

The most notable difference between the Raman spectra of Imid_3_WP·*n*H_2_O and H_3_WPA·*n*H_2_O is found in the high-frequency range where the O–H stretching modes of the aqueous species are found (Figure 2C). While these are peaked at ∼3500 cm^–1^ in the H_3_WPA hydrate, they are markedly red-shifted (by ∼200 cm^–1^) in the Imid_3_WP hydrate. For this new salt, the peak fit analysis reveals two features at 3240 and 3330 cm^–1^ with a fwhm of 100 and 170 cm^–1^, respectively. These peaks are attributed to the water of hydration that experiences stronger hydrogen bonds and a more specific chemical environment than bulk water, as reflected by both the overall red shift of the O–H stretching modes and the longer lifetime (inverse of the fwhm) of the hydrogen bond configurations. (The assignment of these modes to either O–H or N–H stretching modes is nontrivial and complicated by the fact that these are expected to appear at very close frequencies. In addition, in the case of red shift, overlapping may occur, and the typical rule ν(N–H) < ν(O–H) may thus be violated. However, the integrated area of the fitting components found for the Imid_3_WP and the H_3_WPA hydrates gives the ratio 6.08/11.7 (≈0.5), which is close to the ratio 3.7/6 (≈0.6) as expected from considering that H_3_WPA typically comes in the hexahydrate form and that our TGA data suggest a number of water molecules in Imid_3_WP equal to 3.7. Moreover, the few works available in the literature on the vibrational modes of the imidazolium cation report that the symmetric and antisymmetric N–H stretching modes expected at, or below, 3428 cm^–1^ are not observed in the Raman spectrum.^33^ According to the character table of the *C*_2*v*_ point group, which imidazolium belongs to, both symmetric and antisymmetric N–H stretching modes are Raman active; however, these are typically of extremely weak intensity in experimentally recorded Raman spectra.^41^) More precisely, the Raman spectrum of bulk liquid water shows two main features at 3240 and 3445 cm^–1^ with a fwhm of 280 and 290 cm^–1^, respectively. Assuming a linear correlation between the frequency of the O–H stretching mode (of the hydrogen-bond-donating −OH group) and the equilibrium length of the same O–H bond in O–H containing compounds,^42,43^ we can infer that the water of hydration in Imid_3_WP·*n*H_2_O has, on average, O–H bonds with a length of 0.985 Å. Moreover, the shift of the higher frequency component from 3445 cm^–1^ (bulk water) to 3330 cm^–1^ (Imid_3_WP hydrate) is of 115 cm^–1^ and corresponds to an energy difference of 0.014 eV, which is associated with strengthened hydrogen bonds. According to the arguments presented by Agmon around the state of water,^44^ this should correspond to O–H bonds ca. 0.006 Å longer than in bulk water. As a reference, the average O–H bond length in liquid water has been reported to be 0.980 ± 0.019 Å^31^ and the strength of the hydrogen bonds to be 2.6 kcal/mol, that is, ∼0.11 eV.^44^ The relatively weak but sharp peak at 3040 cm^–1^ is of uncertain assignment, but could be attributed to water-bound imidazolium cations and thus to strongly red-shifted N–H stretching modes. This would be consistent with the calculated vibrational frequencies of an imidazole-water dimer (establishing an NH···OH_2_ interaction) that show a difference of ∼300 cm^–1^ between the ν_N–H_ and ν_O–H_ stretching modes.^45^ Another plausible assignment is to the overtone of the ring vibrations of imidazolium found at 1588 cm^–1^, which is consistent with the expected frequency and intensity relations of overtones. (That is, with the relations 3040 · 1.915 cm^–1^ = 1558 cm^–1^ and I_3040_ < I_1588_.)

### Temperature-Dependent Composition

**Figure 3:**
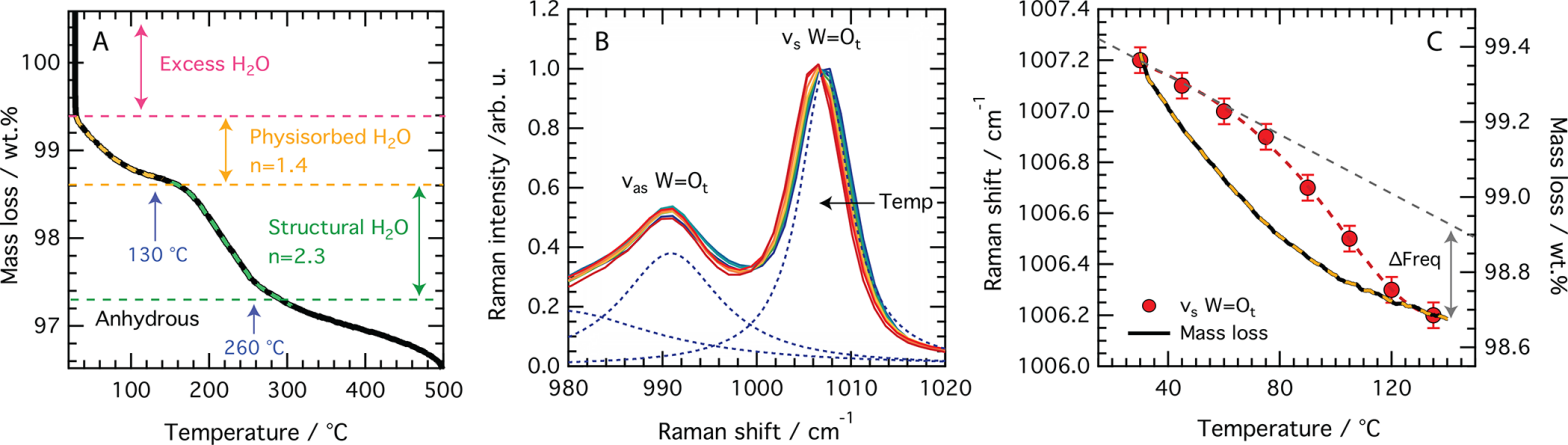
(A) TGA of Imid_3_WP·*n*H_2_O in the temperature range 25–500 °C. The blue arrows indicate the temperature at which the DSC measurements show endothermic transitions. (B) Temperature dependence of the W=O_t_ stretching mode measured by Raman spectroscopy. (C) Comparative plot showing the mass loss event and the Raman shift change in the temperature range 25–145 °C.

Figure 3A shows the TGA curve recorded for the as-prepared Imid_3_WP. This shows that mass loss occurs in several steps and is attributed to excess water, physisorbed water (∼1.4 molecules per Keggin unit), and structural water (∼2.3 water molecules per Keggin unit). (Interestingly, a compound prepared from tungstophosphoric acid and benzotriazole,^46^ albeit not at a strict 1:1 ratio but in a ratio of excess base, revealed a composition with 2 structural water molecules per Keggin unit, which is very similar to the TGA results that we present in this work.) The mass loss observed at temperatures higher than 300 °C is attributed to deprotonation followed by chemical degradation. This dehydration process is corroborated by DSC measurements performed simultaneously to TGA, which reveal endothermic transitions at 132 and 260 °C (marked in Figure 3A with blue arrows, see also Figure S4 in the SI). In Figure S5 of the SI, the TGA curve of the Imid_3_WP hydrate is compared to that of H_3_PWA, which shows much more massive losses. Furthermore, we have experimental evidence (Raman spectra as a function of temperature) that the mass lost upon heating is related to water molecules (see Figure S6 in the SI).

Figure 3B shows the dependence of the frequency of the W=O_t_ stretching vibration as a function of increasing temperature, as measured by Raman spectroscopy using the Linkam Cell. An appreciable red shift is observed, which is opposite to the blue shift of 10 cm^–1^ or more measured by previous authors during thermal treatment of H_3_WPA hydrates.^47−49^ This blue shift was interpreted as being the consequence of a different oxidation state of the tungsten atom due to a changed equilibrium between the protonated aqueous species surrounding the Keggin anion. In all cases, the shift to higher wavenumbers was concomitant with a remarkable decrease in the Raman intensity of the W=O stretching mode, which indicates a change in the primary structure of the heteropolyacid, for example, a lower molecular symmetry or distorted bond angles. By contrast, in the present work, the red shift is not accompanied by any intensity changes, and in addition, the fwhm of the feature at 1007 cm^–1^ is rather stable across the temperature range investigated, that is, 25–145 °C (see Figure S7 of the SI). This demonstrates the thermal stability of the primary and secondary structure of the Imid_3_WP salt, most likely due to the complete proton transfer from the Keggin unit to the imidazolium cation. Hence, differently from the case of the H_3_WPA hydrate where the counterion is in the aqueous phase, in Imid_3_WP, there are distinct ionic interactions that are not dramatically affected by the loss of a few, neutral, water molecules (at least not up to 145 °C). The rate of frequency change for the W=O_t_ stretching mode and the rate of mass loss are compared in a common plot in Figure 3C. Differently from the rate of dehydration measured by TGA, the frequency of the W=O_t_ stretching changes first slowly (up to about 60 °C) and then more rapidly than expected by thermal effects only (see deviation from the linear trend). In other words, the chemical environment of the W=O_t_ bond is mostly affected when the physiosorbed water molecules are all lost, most likely due to a consequent change in the Keggin anion–imidazolium cation interactions. The red shift of the W=O_t_ stretching frequency, observed upon dehydration, is thus the manifestation of slightly longer W=O_t_ bonds. Considering that the P–O bond in the heteropolyanion can shorten upon dehydration,^37^ the observed red shift of the W=O_t_ vibration does not *per se* exclude the contraction of the Keggin unit as a whole.

### Proton Conductivity

**Figure 4:**
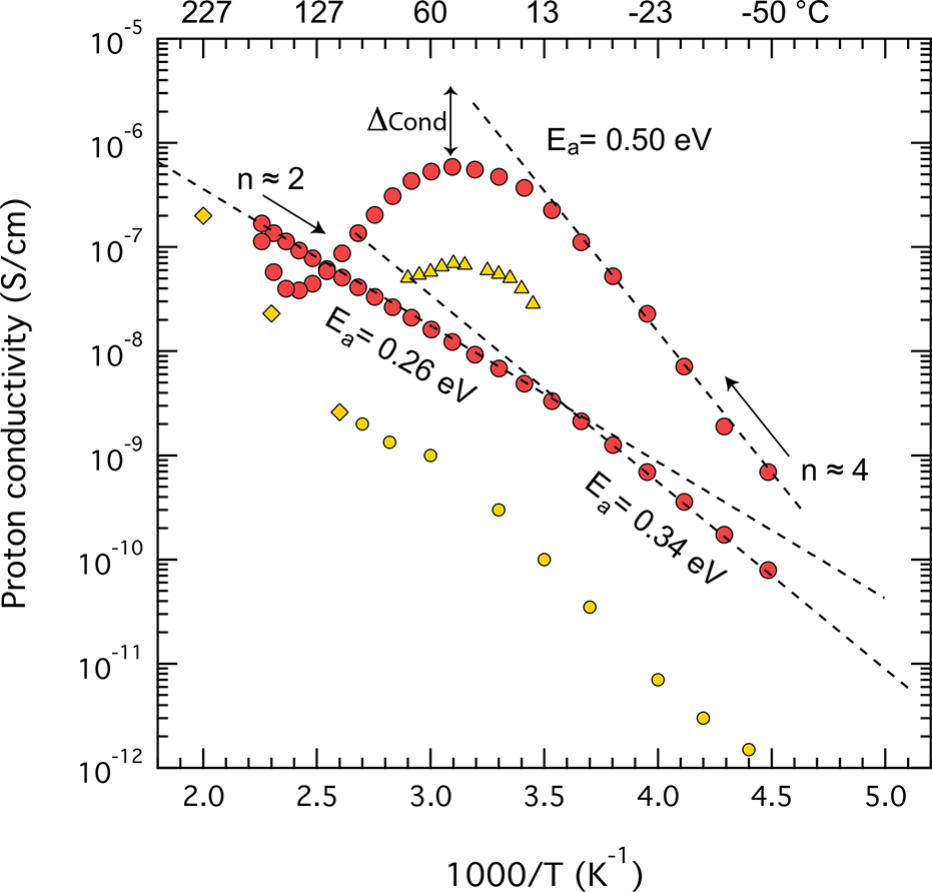
Arrhenius plot of the proton conductivity measured for the salt hydrate Imid_3_WP·*n*H_2_O (red filled circles) upon heating and subsequent cooling. The conductivity values for the reference material H_3_WPA·*n*H_2_O (n ≈ 0) measured by us in this study (yellow circles) and reported by Tjapkin et al.^5^ (yellow diamonds) are also shown for comparison as well as the conductivity values reported by Mioc et al.^8^ for MgHWPA·nH_2_O (*n* = 12) (yellow triangles). Dashed lines are fits to the experimental data.

The effect of temperature, and thus composition, on the proton conductivity of Imid_3_WP·*n*H_2_O is shown in Figure 4. The Arrhenius plot shows an initial rapid increase of conductivity with temperature (see direction of the arrow) that is associated with an activation energy, *E*_a_, of 0.50 eV. This value has been obtained from the low-temperature region of linear behavior and by fitting with the relation σ = σ_0_·e^–*E*_a_/*kT*^. The magnitude of *E*_a_ suggests some contribution from the vehicular mechanism of charge transport between −50 °C and +20 °C. Within this temperature range, and according to the TGA results, the salt hydrate contains 4 water molecules per structural unit. At higher temperatures, however, a continuous decrease in conductivity is observed, from about 20 °C up to 145 °C (i.e., between 3.4 and 2.4 in the 1000/T axis) that according to the TGA data discussed above is due to partial dehydration. This results in a new stoichiometric composition and a salt hydrate that at 145 °C contains only ∼2 water molecules per Keggin unit. (Or <2, considering that TGA data are obtained under continuous heating while we use longer equilibration times during the conductivity experiments.) At this composition, the salt can be considered as pseudoanhydrous.

To better understand the relation between local structure and proton conductivity in the range 25–145 °C, we have compared the deviation of conductivity from the value expected without dehydration (Δ_Cond_) with the deviation of the W=O_t_ stretching frequency from the value expected under thermal effects only (Δ_Freq_), see the double arrows in Figures 4 and 3C, respectively. These deviations, extracted from two independent measurements, are shown in a comparative plot in Figure S6 of the SI. It is notable that these quantities depend on temperature in a very similar manner, with a higher rate of change above 60 °C. This demonstrates a strong composition–dynamic correlation and suggests that physisorbed water molecules, responsible for screening the anion–cation interactions, are also somehow involved in the vehicular transport of protons.

Upon the following cooling scan (i.e., from +145 to −50 °C), the conductivity shows a linear dependence on inverse temperature and is associated with a reduced activation energy of only 0.26 eV. This value is comparable to that measured for other proton conducting Keggin-ion based salts and acids, such as MgHWP·12H_2_O (0.20 eV for temperatures between 32 and 50 °C; 0.28 eV for temperatures below 32 °C),^8^ BaHWP·7H_2_O (0.27 eV for temperatures between 10 and 50 °C),^9^ and H_3_WPA·29H_2_O (0.23 eV around 40 °C).^5^ (Note: For the H_3_WPA·*n*H_2_O hydrate, the activation energy of proton transport increases upon dehydration; from 0.23 eV for 29 water molecules up to 1.07 eV for 6 water molecules.^5^) The activation energy of 0.26 eV measured in this work is also very close to that measured for proton conduction in liquid water (0.22 eV if the mechanism of proton hopping is described by the consecutive cleavage of *two* hydrogen bonds, 0.11 eV each^44^) and liquid imidazole (0.25 eV;^50^ 0.23^51^), values that are typically associated with the energy needed to break hydrogen bonds in these compounds.^44,50,51^ While discussing the activation energy of mobile protons in solid ionic materials, it is also relevant to mention the values measured for recently developed materials based on imidazole, that is, an activation energy of 0.35 eV for imidazole-aluminum phosphate hybrids at temperatures above 84 °C,^52^ between 0.50 and 0.15 eV for imidazolium-malonate between 5 and 84 °C,^53^ and 1.5 eV for imidazolium-benzoate between 45 and 70 °C.^54^ A class of materials that display a similar dependence of proton conductivity on temperature (i.e., similar to that shown in Figure 4) is that of proton conducting ceramics,^55^ which include doped perovskites and simple metal oxides. These materials display a distinct temperature dependence of conductivity as a direct effect of dehydration and also show behaviors that relate to grain boundary effects. Within this family of materials, the doped perovskite BaZr_0.9_Y_0.1_O_3_ displays, for example, a proton conductivity of ∼3 × 10^–6^ S/cm at 50 °C, a value that decreases with an increasing temperature reaching a minimum at 150 °C.^55^ The activation energy for this perovskite has been reported to be 0.36 eV for the bulk and 0.76 eV at grain boundaries. The aspects exposed above demonstrate that in Imid_3_WP·2H_2_O, that is, the pseudoanhydrous material presented in this work, protons are transported by hopping events along hydrogen bonds. However, it remains unclear whether these events occur on the surface of a Keggin anion primarily (*intra*molecular), or also include *inter*molecular hopping. The nature of the conduction pathways will be discussed further down through the analysis of results achieved from quantum calculations and MD simulations.

The activation energy of proton transfer is slightly higher, and equal to 0.34 eV, at temperatures below −3 °C (Figure 4). Since this transition temperature coincides with that of an endothermic peak recorded in the DSC trace (see Figure S4 in the SI), this change is attributed to a local liquid–solid transition of the water–water or water–imidazolium phase. The difference in activation energy of 0.08 eV (= 0.34–0.26 eV) is comparable to the enthalpies of crystallization for water (i.e., 6 kJ/mol or 0.06 eV) and imidazole (12.8 kJ/mol or 0.13 eV) and can thus be associated with the strengthening of NH···O/N and/or OH···O/N hydrogen bonds upon cooling. Furthermore, the reduced activation energy upon dehydration, that is, the change from 0.50 eV for Imid_3_WP·4H_2_O to 0.26 eV for Imid_3_WP·2H_2_O, is consistent with an increased contribution from proton hopping events and with the expected shift of the proton from the imidazolium cation toward the WP anion upon loss of water.^56^

It is notable that despite the lower degree of hydration (i.e., n≈4), Imid_3_WP shows proton conductivities higher than any other salt prepared from H_3_WPA (for instance by substituting one or more protons with positively charged ions like Ba^2+^ or Mg^2+^),^8,9^ as shown by comparing to a selection of already reported data, reproduced in Figure 4 for ease of comparison. This observation is even more important considering that the proton conductivity in the referred materials was measured under a relative humidity of 30% or higher, while our data were obtained under strictly anhydrous conditions (i.e., under flow of dry N_2_ gas). This is a very relevant insight since in all the related ionic compounds previously investigated, the conductivity has shown to depend dramatically on the number of water molecules present in the hydrate, with conductivity drops of several orders of magnitude upon dehydration.^5^ To summarize, the Arrhenius plot in Figure 4 shows that the new salt hydrate Imid_3_WP·*n*H_2_O is superior to both hydrated and anhydrous H_3_WPA·*n*H_2_O, and compounds derived thereof, in a wide range of temperatures.

### Molecular Orientation and Local Dynamics

**Figure 5:**
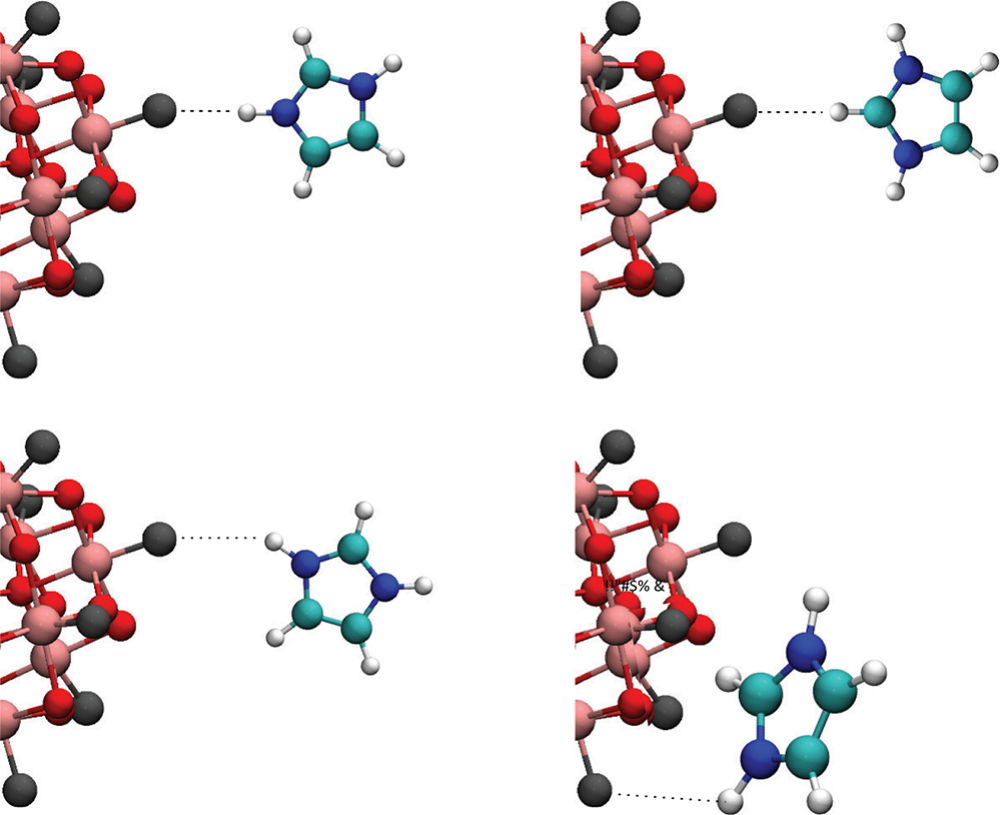
Most probable molecular orientations and types of hydrogen bonds that can be established between the imidazolium cation and the WP anion.

Additional important insights on the mechanism of proton conduction can be achieved from understanding the local orientation of molecules and the crystalline structure of Imid_3_WP·*n*H_2_O. Considering that the most acidic protons in imidazolium are those on the nitrogen sites, it is plausible to assume that hydrogen bonds would preferably form between the −N^1,3^H^+^ group and the oxygen atoms of the tungstophosphate anion. This would be in great accordance with the bonds formed in several types of imidazolium-carboxylate salts.^53,54,57,58^ Nevertheless, this insight does not preclude any of the possible structures shown in Figure 5, which shows different possible orientations of the imidazolium cation with respect to the Keggin anion’s surface. In this context, it should be considered that upon protonation of imidazole, the C^2^H proton becomes more acidic and thus also a possible site for the formation of hydrogen bonds. The corresponding C^2^H^+^···O^–^ hydrogen bonds, however, are expected to be weaker and less linear than those found in the N^1,3^H^+^···O^–^ configuration.

**Figure 6:**
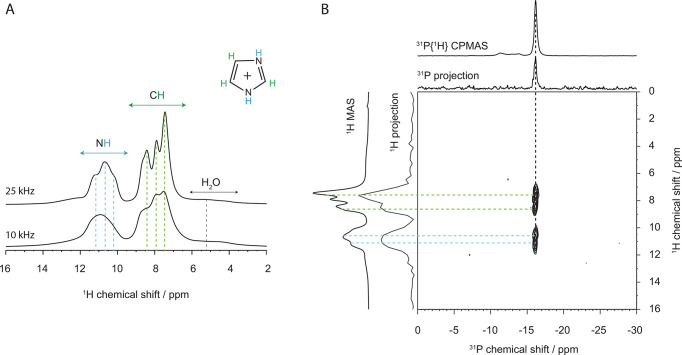
(A) ^1^H MAS experiments performed in the solid state with spinning rates of 10 and 25 kHz. (B) ^31^P{^1^H} HETCOR NMR spectra collected at 15 kHz with 800 μs of contact time.

Solid-state ^1^H NMR spectra recorded at two different spinning rates are shown in Figure 6A and reveal distinct signatures assigned to N^1,3^H and C^2,4,5^H protons. The signature of water is found between 6 and 4 ppm and shows a broader shape. One can notice that the NH feature contains several components, reflecting a diversity of chemical environments for the NH protons and thus a certain degree of structural disorder for the imidazolium cations. In fact, a careful peak fitting analysis of the chemical shift range 16–9 ppm reveals an additional broad contribution and hence the copresence of less mobile −NH species (see Figure S9a in the SI). This finding is very similar to the results presented by Rachocki et al.^58^ and Lawniczak et al.,^53^ that is, that the imidazolium cations in imidazolium-oxalate and imidazolium-malonate exist as both slow and fast molecules, in other words as both ordered and disordered species. The real space proximity of molecular species has been resolved by solid-state NMR spectroscopy, performing advanced 2D HETCOR ^31^P{^1^H} experiments (Figure 6B and Figure S9b of the SI). The main ^31^P peak found at −16.00 ppm is consistent with the assignment presented by de Oliveira et al.,^37^ which also suggests that the much weaker signatures observed to the left of the main peak could be due to traces of less hydrated polyanion species. Interestingly, the resulting heteronuclear correlation pattern reveals a proximity to the Keggin anion of both N^1,3^H and C^2,4,5^H protons, indicative of a flat orientation of the imidazolium cations with respect to the Keggin ion surface.

**Figure 7:**
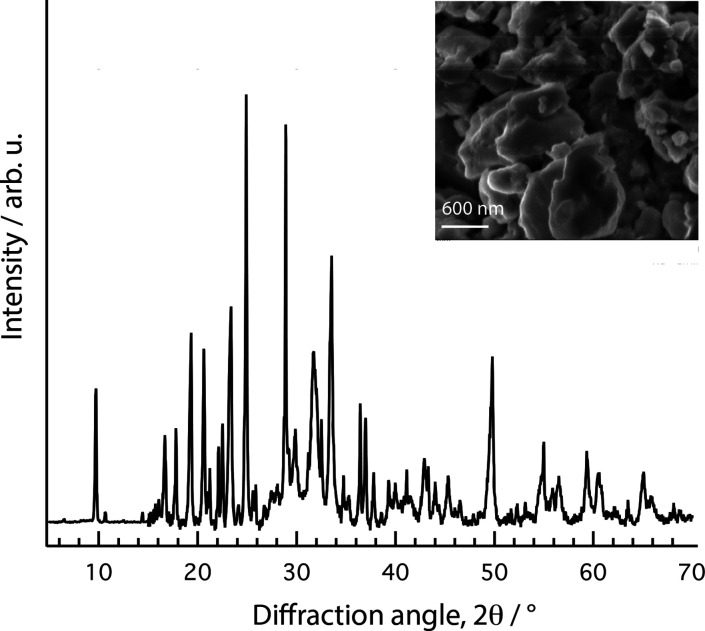
XRD pattern (main) and SEM image (inset) of the Imid_3_WP salt hydrate.

This arrangement allows for a variety of NH^+^···O^–^ bonds and distances and is in agreement with the multicomponent shape of the N^1,3^H feature discussed above for the 1D ^1^H NMR spectrum. Most importantly, as a consequence of the flat arrangement, short P–P (or Keggin anion–Keggin anion) distances can be established. This is in fact what the XRD data also indicate, through the low-angle diffraction peak at 9.7°, Figure 7, that corresponds to a repeating distance of 9.1 Å. This is shorter than expected from the Keggin ion nominal radius of 5.2 Å,^59^ but not in contrast to the finding that WP anions can come to very close contact without energy penalty when water molecules bridge between O_t_ and O_b_ sites of adjacent Keggin units, thus compensating for the Coulombic repulsion.^59^ In the computational study of Chaumont et al.,^59^ it is also emphasized that the closest P–P distance strongly depends on the size and charge distribution of the countercation. Relevant to this discussion are the results retrieved for ethanol solvated H_4_[SiMo_12_O_40_], showing that ethanol and water molecules occupy interstitial sites between Keggin anions, establishing hydrogen-bond interactions to the oxygen atoms of the anion, and can easily be removed at higher temperatures without significant structural changes.^60^ Similarly, a detailed MD simulations study has shown that WP anions can come in very close contact, overcoming electrostatic repulsion, when ethylimidazolium is the countercation.^61^ This study also shows how hydrating water molecules then prefer to reside in the cavities between the O_t_ and the O_b_ oxygens of the Keggin anion. From the collected XRD data and using the Scherrer formula, the size of the crystallites has been estimated to be around 600 Å (i.e., 60 nm), while morphological SEM images reveal particles around 1 μm in size. Other salts, based on the tungstophosphoric anion and cations such as magnesium, potassium, cesium, or ammonium, have shown spherical particles with a diameter of ∼400 nm,^8^ monoclinic crystals with a size of 10 μm,^10^ or larger crystals with a less defined geometry.^7^ For a more detailed structural information on Imid_3_WP·*n*H_2_O, however, future studies should also include the use of neutron powder diffraction and single crystal X-ray diffraction. Additional insight into the orientation of the imidazolium cations around the Keggin anions will also be provided by analyzing the MD predictions, see Computational Insights section further down.

The local dynamic of different protons, such as rotational motions and/or fast local exchanges, has been studied by relaxation solid-state NMR measurements. The values of the relaxation time, *T*_1_, are found between 1.6 and 1.9 s at 20 °C but increase with temperature (Figure S9 of the SI). The increase of *T*_1_ with temperature is more pronounced for N^1,3^H and C^2^H protons (if compared to C^4,5^H protons), indicating that these are involved in faster local dynamics, especially the N^1,3^H protons. The activation energy of these dynamical events has been calculated, invoking the BPP theory, and from the Arrhenius plots of log(*T*_1_), it is found to be in the range 4–6 kJ/mol, that is, approximately 0.04–0.06 eV. These values are much smaller than, for example, those reported for salts obtained from imidazole and a carboxylic acid, for which the activation energy for rotation was found to be 0.22 eV and assumed to describe the 180° flip of the whole imidazolium molecule around its *C*_2_ axis.^58^ The smaller values of *E*_a_ found from relaxation NMR measurements in this study may thus relate to proton exchange events rather than a full molecular reorientation. Moreover, since the range 0.04–0.06 eV is well below the *E*_a_ values estimated from impedance spectroscopy (*E*_a_ ≈ 0.26–0.50 eV), we conclude that the underlying local dynamic (supposedly localized exchange reactions) is not rate limiting for the long-range proton transfer in the Imid_3_WP·*n*H_2_O hydrate. Hence, breaking of the strong hydrogen bonds is likely the most energetically demanding step for the proton motion to occur.

### Computational Insights

**Figure 8:**
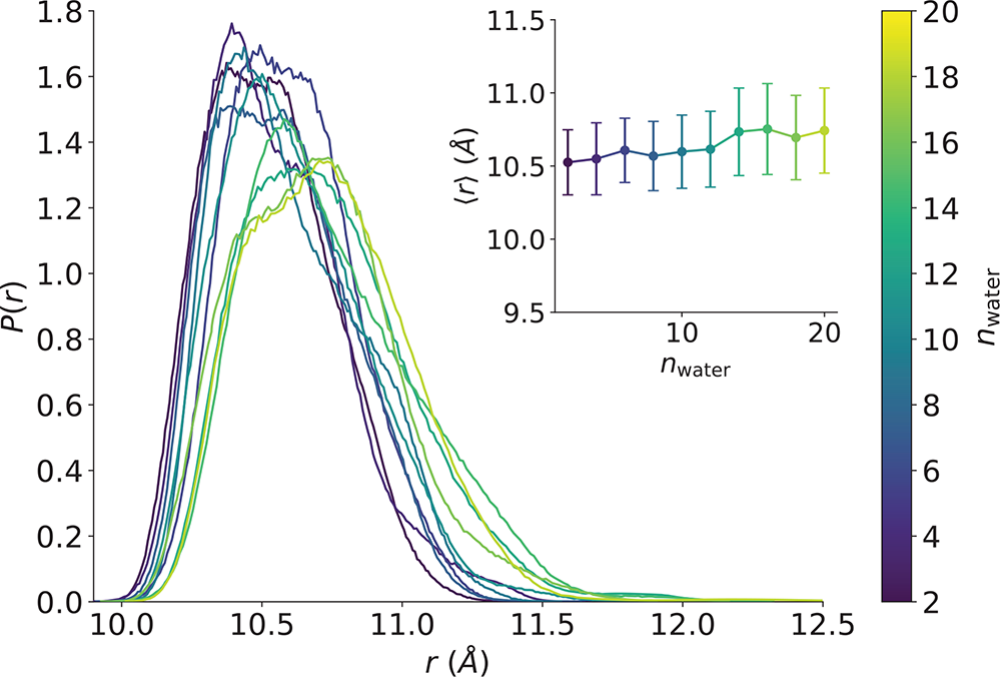
Distribution of closest distances between Keggin anions (i.e., P–P distances) for all the simulated degrees of hydration (represented by the number of water molecules, *n*). The average of these distributions, along with the respective standard deviation, is shown in the inset plot.

More information about the fate of water, which could not be resolved by solid-state NMR, could be retrieved from MD simulations. First, we calculated the distribution of P–P distances, that is, the distances between one Keggin anion and its closest neighbor, to compare with the results from XRD data. These distributions, calculated for all the simulated degrees of hydration, as well as their average and standard deviation are represented in Figure 8. As one could expect, the average distance between Keggin anions increases slightly with the amount of water, see the inset plot of Figure 8. Moreover, the distribution gets sharper for lower values of *n* (i.e., a lower degree of hydration), reflecting a more ordered structure. In addition, the average P–P distance approaches the value of 10.55 Å, which is comparable to twice the nominal radius of the WP anion (5.2 Å).

**Figure 9:**
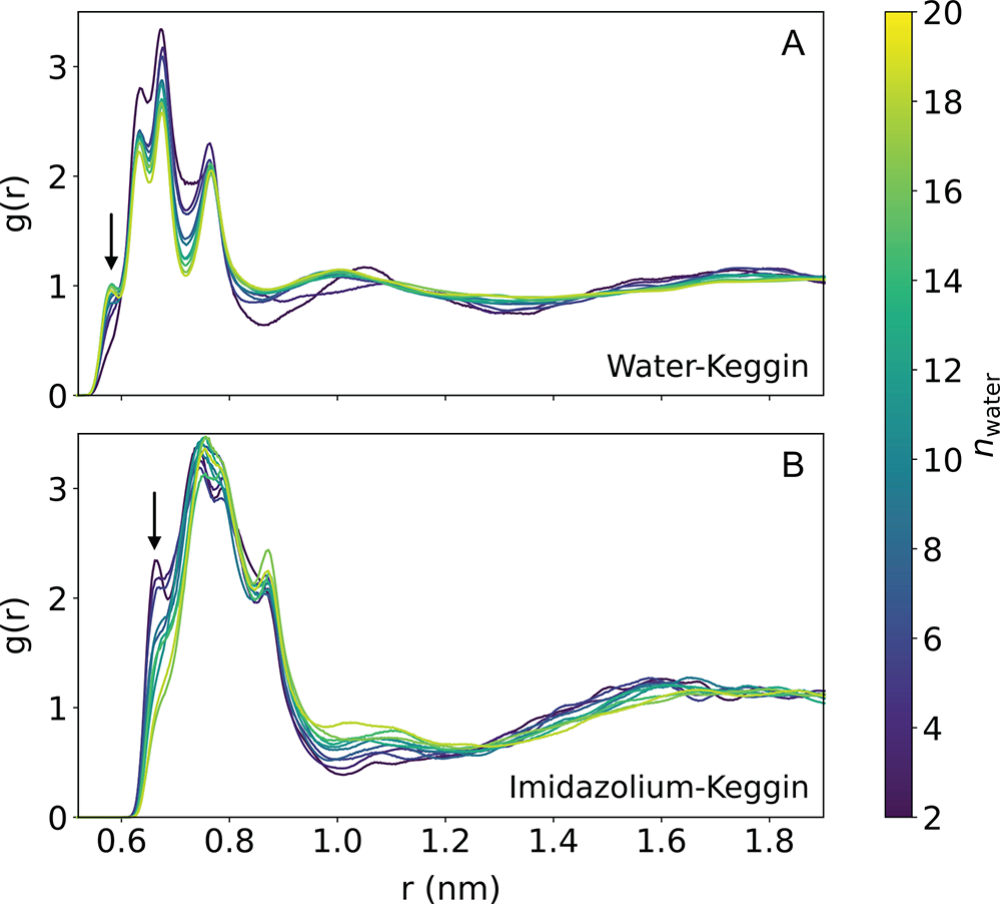
RDFs of water molecules (A) and imidazolium cations (B) around the Keggin anions, for all the simulated degrees of hydration.

The distribution of imidazolium cations and water molecules around the Keggin anions has also been studied, using the radial distribution functions (RDFs) as summarized in Figure 9. The molecular centers of mass were used to compute the distances for this analysis. The RDFs clearly show the existence of multiple adsorption sites and molecular coordinations around the large Keggin anion. Indeed, as the salt’s degree of hydration (*n*) increases, the intensity of the first peak in the imidazolium distribution decreases, while the peak intensity associated with the closest adsorption site of water increases (see peaks indicated by an arrow in Figure 9). In the imidazolium distribution plot, this peak corresponds to cations whose center of mass is very proximate to the Keggin unit, most likely to imidazolium cations oriented flat with respect to the anion’s surface.

**Figure 10:**
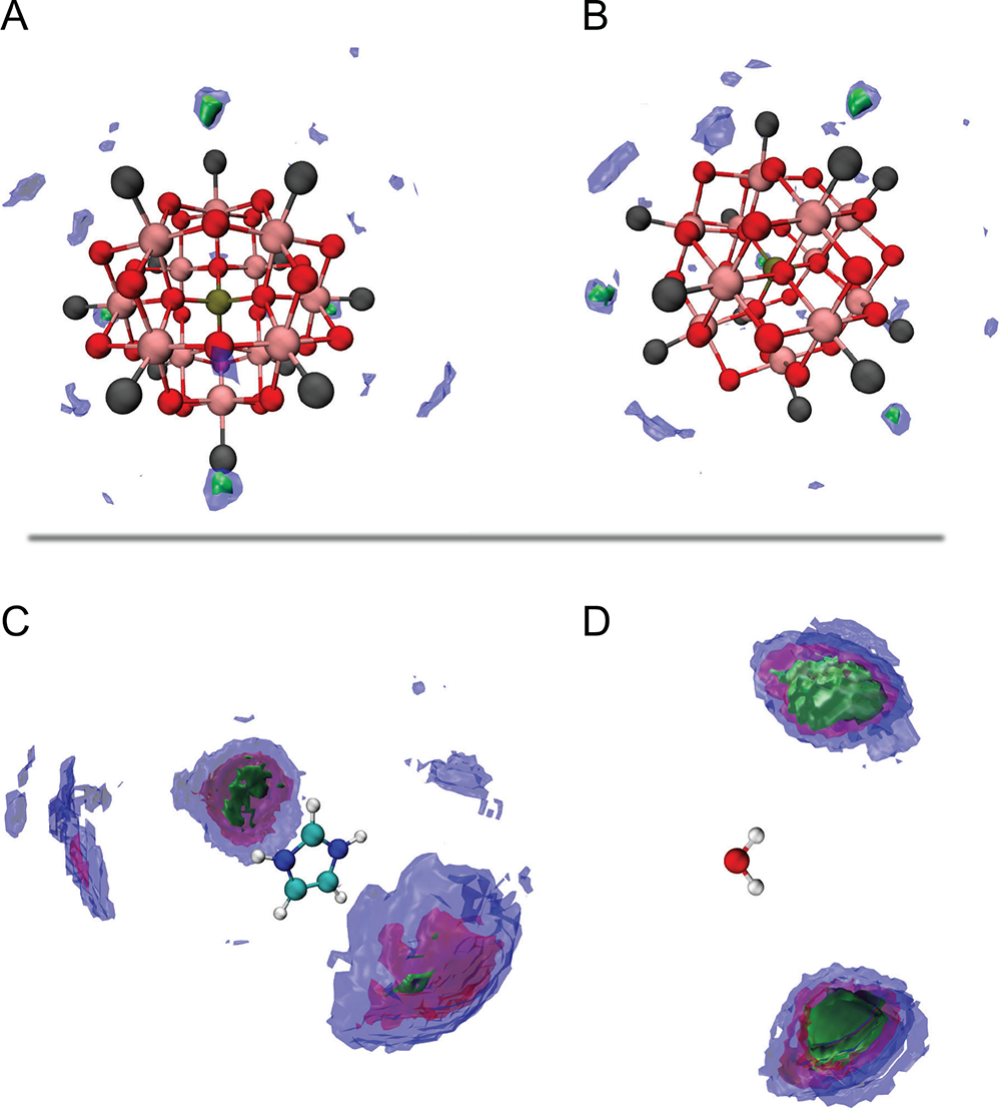
SDFs of water molecules around Keggin anions from two perspectives (A and B) as well as of Keggin anions around either imidazolium cations (C) or water molecules (D). The cutoff values are chosen for a better visualization and are referred to the value corresponding to a random distribution, ρ_bulk_. In the top plots, green and blue correspond to 41.7 and 16.7 times ρ_bulk_, while in the bottom plots green, red, and blue correspond to 16.5, 12.6, and 8.9 times ρ_bulk_, respectively. For the case of water around Keggin anions, this value is calculated assuming that water can only exist in the space not occupied by the anions. In (A and B), the O_t_ atoms are colored in black.

To get a better insight into the mentioned adsorption sites and molecular orientations, the spatial distribution functions (SDFs) of selected molecular species were calculated for all water concentrations. As a representative case, the results from the simulations for *n* = 4 are shown in Figure 10. If the distribution of water around the Keggin anion is analyzed, a very interesting phenomenon is observed, that is, there are four distinct adsorption sites for all levels of hydration. These sites are in the middle of the four triangles formed by the *O*_t_ oxygens and are thus defined by the intrinsic symmetry of the Keggin structure. Although not explicitly shown here, as the water content increases, these sites remain occupied, while the additional water molecules get randomly distributed around the anions. Moreover, we find that water molecules are oriented with one hydrogen atom pointing toward the center of the anion, such that the formation of hydrogen bonds is favored. Finally, analyzing the distribution of anions around the imidazolium cations, we can distinguish between two populations. Some of the cations are oriented with the N–H group pointing toward the center of the anion forming hydrogen bonds, while others lay flat on the anions’ surface. A quantitative estimation of these two configurations is given in Figure S8 and shows, in agreement with results from the RDFs, that as the number of water molecules per anion increases (and hence the distance between Keggin anions), the proportion of cations flatly oriented decreases. In other words, in the most densely packed configuration, the imidazolium cations adopt the orientation that minimizes the distance between anions, that is, the flat orientation. The SDFs calculated for cations around the anions did not reveal any specific adsorption site, which, unlike the case of water, suggests a random spatial distribution around the Keggin structure. This structural scenario may translate into the coexistence of imidazolium cations with different mobility, those establishing strong hydrogen bonds likely being the less mobile. This would be in line with the results from solid-state NMR discussed above.

**Figure 11:**
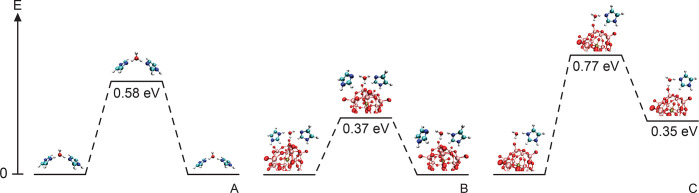
Schematic representation of the energy for the most relevant states along the proton transfer paths. The values of the energy are referred to the initial state which corresponds to the situation where the proton is bonded to the imidazole molecule.

We have also studied the proton transfer energy barriers by means of DFT simulations, calculating the energy difference between the state before the proton leaves the donor species and the intermediate state, in which the proton forms an hydronium ion. As experimentally shown, the protonated species in the studied system is the imidazolium cation, which thus plays the role of the proton donor. On the other hand, as evidenced by structural characterization, water molecules adsorbed around the Keggin anions act as a bridge for proton transfer between imidazolium cations and imidazole molecules. Of relevance for our calculations is one work of Kaila and Hummer, reporting that the energy barrier for proton transfer, considering imidazolium-based molecules mediated by one water molecule, is around 0.60 eV.^63^ We have followed the same procedure to calculate the energy barrier in the case of proton transfer between imidazolium cations and imidazole molecules (see Figure 11 for a schematic representation of the simulated systems). We obtained an energy barrier of 0.58 eV (Figure 11A), which agrees well with the results referred to above. To study the effect of the Keggin anion on the energy barrier, we reproduced the same calculation but in the presence of one proximate WP unit, including a coordination between this and water as it happens in the MD simulations. In this scenario, we observed a remarkable decrease of the energy barrier, down to 0.37 eV (Figure 11B). In other words, the Keggin anion acts as a catalyzer in the proton transfer process through this simulated pathway. Another possible transfer path could be from one imidazolium cation to the Keggin anion, mediated by an adsorbed water molecule. We have calculated the energies of the states in this proton transfer path (Figure 11C), and it can be seen that the barrier is as high as 0.77 eV. Altogether these results indicate that the proton is unfavorably residing on the hydronium ion or on the WPA acid, but rather prefers to reside on the imidazolium cation, which is congruent with the Raman spectroscopy results showing that imidazole is a stronger base than water and hence the protonated species in this salt hydrate. Also, they show that the presence of the WP anion facilitates proton transfer between imidazole species mediated by water molecules (see Figure 11B).

## 4 Conclusions

We have pioneeringly tuned the properties of a heteropolyacid-based solid-state proton conductor by including into the structure both water and imidazole, a common precursor to ionic liquids, showing that the conductivity and stability of anhydrous polyoxometalate electrolytes can be significantly improved. More specifically, we have studied the structure and dynamics in a salt hydrate that, to the best of our knowledge, has never been reported before, that is, Imid_3_WP·*n*H_2_O. This material is obtained from reacting 12-tungstophosphoric acid, that is, H_3_PWA, with the base imidazole in an aqueous solution. By a combination of appropriately chosen experimental methods, we find that this material is chemically stable upon increased temperature (up to at least 150 °C) and displays an intrinsically high proton conductivity, both in the hydrated (*n* ≈ 4) and in the pseudoanhydrous (*n* < 2) state. This proton conductivity is higher than that measured in other proton conductors based on or derived from tungstophosphoric acid, despite the lower degree of hydration and no humidity during the conductivity measurements. These aspects make this material interesting as a new solid-state proton conductor. For Imid_3_WP·*n*H_2_O with *n* = 0–2, we report conductivity values of 10^–7^ S/cm at approximately 130 °C, the highest ever measured for salts or acids derived from H_3_PWA at anhydrous conditions. Moreover, from the Arrhenius plot of conductivity, a low activation energy of 0.26 eV is estimated, indicating a mechanism of proton transfer that includes hopping assisted by hydrogen bonds. MD simulations reveal a local structure that confirms the experimental results from solid-state NMR, that is, that the imidazolium cations can adopt two orientations and that they are preferably flat with respect to the Keggin anion’s surface at low levels of hydration. An additional and exclusive detail revealed by MD simulations is that water molecules occupy the triangular cavities defined by the O_t_ oxygens of the Keggin unit. This minimizes the total volume and is congruent with the very short P–P distances measured and calculated for neighboring Keggin units. By virtue of being a new solid-state proton conductor at anhydrous conditions and intermediate temperatures, the salt hydrate Imid_3_WP·*n*H_2_O generates interest for use in devices like proton sensors, fuel cells, or supercapacitors. Nevertheless, we foresee that for use in real applications and property optimization, future studies should be directed to the performance also at higher temperatures (e.g., in the melt), to the effect of mixing with a polymer such as PEO (known to be compatible with proton conduction and able to provide a low *T*_g_ for enhanced ionic dynamics), and/or to understanding the effect of grains’ size (within the nanometer scale) and grain boundaries on the conduction of protons.

## Supporting Information Available

Additional and complementary Raman and infrared spectra; extended versions of DSC and TGA curves; results from peak fit analyses of Raman spectra and conductivity data; additional solid-state NMR spectra and their analysis; additional computational results on molecular orientation (PDF)
