# Supplementary material for: A New Solid-State Proton Conductor: The Salt Hydrate Based on Imidazolium and 12-Tungstophosphate
Source: J Am Chem Soc. 2021 Aug 18;143(34):13895–907. doi: 10.1021/jacs.1c06656 (PMC8414554; doi:10.1021/jacs.1c06656)
Supplement: Supplementary file 1 — ja1c06656_si_001.pdf [file ja1c06656_si_001.pdf]

SUPPORTING INFORMATION

A New Solid State Proton Conductor: The Salt Hydrate Based on Imidazolium and 12-Tungstophosphate[†]

Anna Martinelli,[a*] José M. Otero-Mato,[b] Mounesha N. Garaga,[c] Khalid Elamin,[a] Seikh Mohammad Habibur Rahman,[a] Josef W. Zwanziger,[c] Ulrike Werner-Zwanziger,[c] and Luis M. Varela[b]

[a] Department of Chemistry and Chemical Engineering, Chalmers University of Technology, Gothenburg, Sweden
[*] E-mail: anna.martinelli@chalmers.se (corresponding author)
[b] Department of Physics, University of Santiago de Compostela, Santiago de Compostela, Spain
[c] Department of Chemistry, Dalhousie University, Halifax, Canada

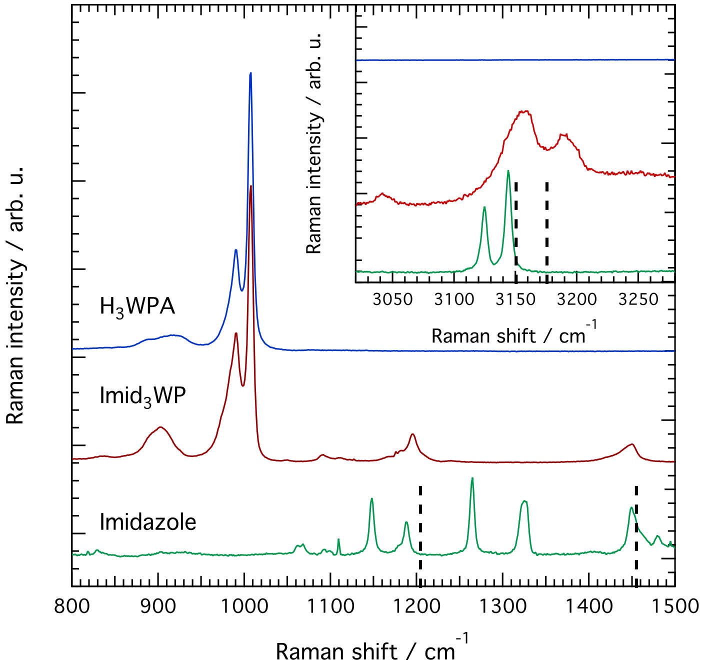

Figure S1. Raman spectra collected at room temperature for H_3WPA, $Imid_3WP$ and neutral (un-protonated) imidazole. The high-frequency range of the spectra, where $C^{2,4,5}$–H stretch modes appear, is shown in the inset. The vertical dashed lines indicate the frequency of vibrational modes calculated for the imidazolium cation.[1]

References

1 Majoube, M. and Henry, M. and Chinsky, L. and Turpin, P.Y. Preresonance Raman spectra for imidazole and imidazolium ion: interpretation of the intensity enhancement from a precise assignment of normal modes. *Chemical Physics*, **1993**, *169*(2), 231–241.

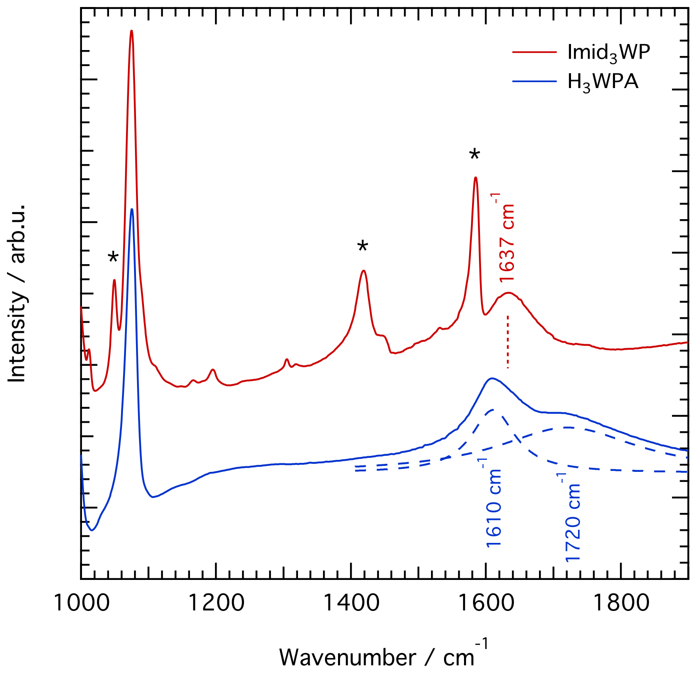

Figure S2. Infrared spectra collected at room temperature for the $Imid_3WP$ and the H_3WPA hydrates, in the region of the H-O-H bending modes. Asterisks indicate vibrational modes arising from the imidazolium cation.

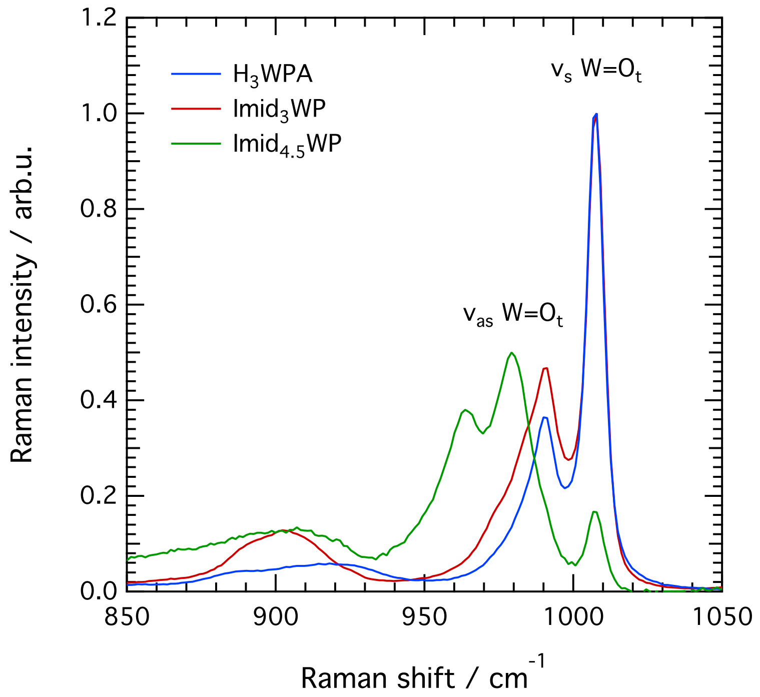

Figure S3. Raman spectra recorded at room temperature for $Imid_x WP$ salt hydrates where x is equal to 0 (i.e. the reference H_3WP salt), 3 (acidic environment of the Keggin ion) and 4.5 (basic environment of the Keggin ion).

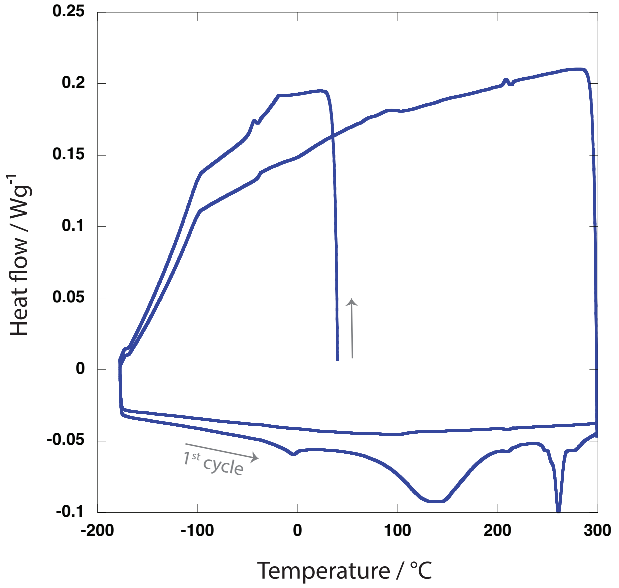

Figure S4. DSC traces recorded for the $Imid_3WP$ salt hydrate, showing the first and second cooling/heating cycles.

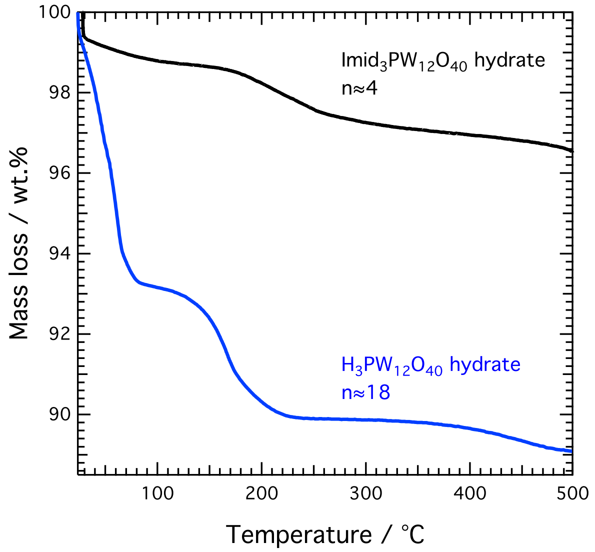

Figure S5. TGA curves recorded for the reference compound H_3WPA (blue trace) and the salt hydrate $Imid_3WP$ (black trace).

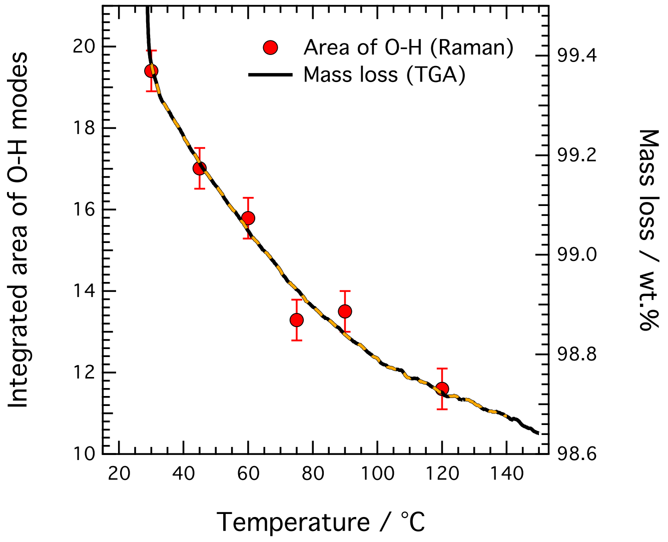

Figure S6. Integrated area of the vibrational modes attributed to O-H stretching, i.e. to water (Raman), and mass loss (TGA) as a function of increased temperature.

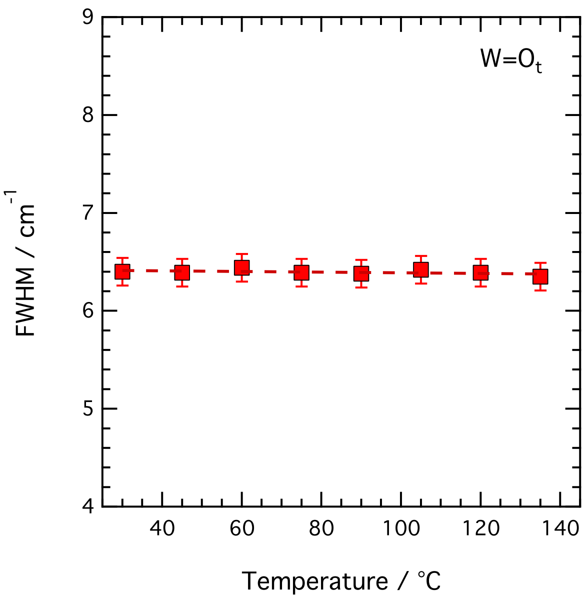

Figure S7. Full Width at Half Maxmimum (FWHM) of the W=O_t symmetric stretching mode as a function of increased temperature, and thus dehydration of the $Imid_3WP$ hydrate.

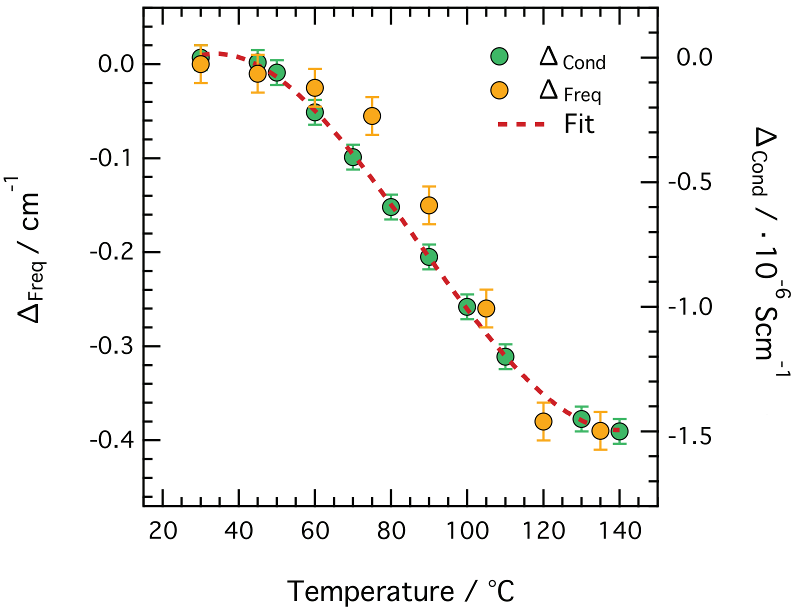

Figure S8. Deviation from the case of thermal effects only of the frequency of the W=O$_t$ symmetric stretching mode (yellow symbols) and the deviation of proton conductivity from the case of no dehydration (green symbols), for the $Imid_3WPA·nH_2O$ hydrate and as a function of temperature.

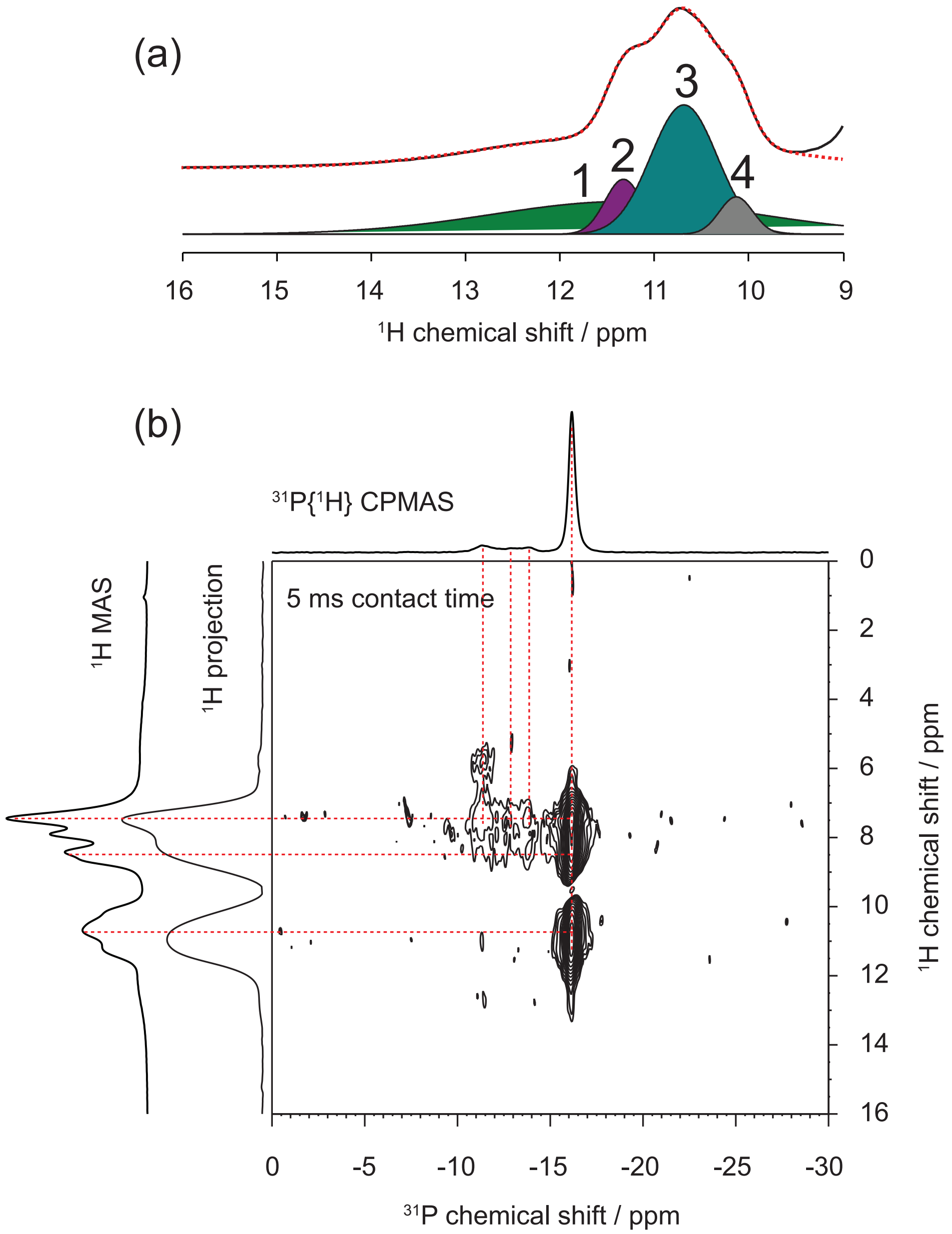

Figure S9. (a) Deconvolution of the 1D ^{1}H MAS NMR spectrum (NH region) fitted using the DMFIT software [Ref: Massiot et al., Magnetic resonance in chemistry, vol. 40, pp70-76 (2002)], highlighting the presence of different NH species. (b) 2D ^{31}P^{1}H HETCOR NMR spectrum collected on a 400 MHz Bruker NMR spectrometer with the contact time set to 5 ms.

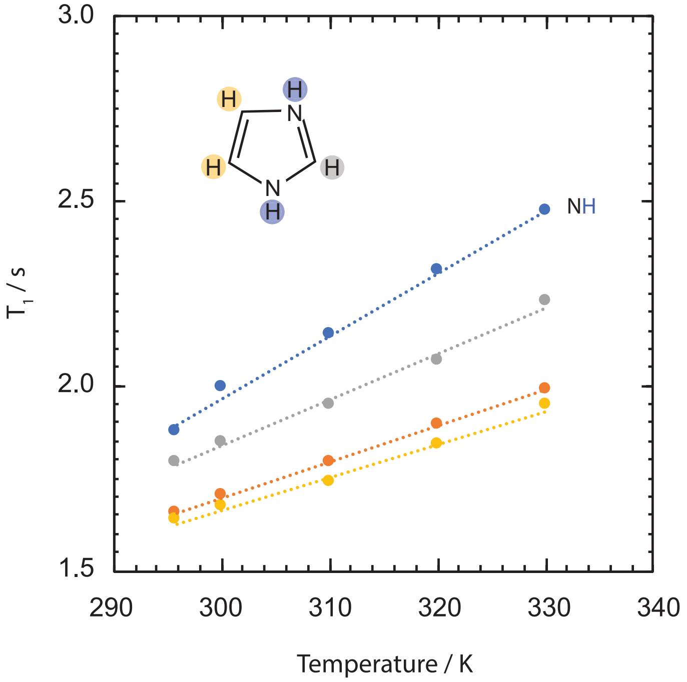

Figure S10. ^{1}H spin-lattice relaxation time (T1) measured as a function of temperature (23 °C – 57 °C) on a 400 MHz spectrometer by using a saturation recovery pulse sequence. The ^{1}H signal was collected over 16 increments with the delay, which allow for spins to relax, varied up to 15 s. The sample was spun at 20 kHz MAS.

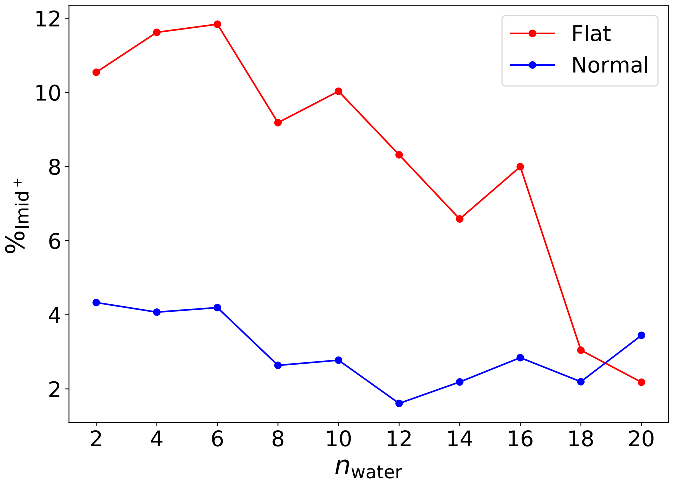

Figure S11. Fraction of imidazolium cations oriented flat or normal with respect to the surface of the Keggin anion, for all the simulated hydration levels.
